# NF-κB-Induced R-Loops and Genomic Instability in HTLV-1-Infected and Adult T-Cell Leukemia Cells

**DOI:** 10.3390/v14050877

**Published:** 2022-04-23

**Authors:** Chou-Zen Giam, Nagesh Pasupala

**Affiliations:** Department of Microbiology and Immunology, Uniformed Services University of the Health Sciences, Bethesda, Rockville, MD 20814, USA; nagesh.pasupala.ctr@usuhs.edu

**Keywords:** human T-cell leukemia virus type 1, adult T cell leukemia, tax, NF-κB, R-loop, DNA double-strand breaks, senescence, genomic instability, nucleotide excision repair

## Abstract

Human T-cell leukemia virus type 1 (HTLV-1) is a human delta retrovirus that causes adult T-cell leukemia/lymphoma (ATL) in 3–5% of the infected population after decades of clinical latency. HTLV-1 Tax is a potent activator of IKK/NF-κB and a clastogen. While NF-κB activities are associated with cell survival and proliferation, constitutive NF-κB activation (NF-κB hyperactivation) by Tax leads to senescence and oncogenesis. Until recently, the mechanisms underlying the DNA damage and senescence induced by Tax and NF-κB were unknown. Current data indicate that NF-κB hyperactivation by Tax causes the accumulation of a nucleic acid structure known as an R-loop. R-loop excision by the transcription-coupled nucleotide excision repair (TC-NER) endonucleases, Xeroderma pigmentosum F (XPF), and XPG, in turn, promotes DNA double-strand breaks (DSBs). NF-κB blockade prevents Tax-induced R-loop accumulation, DNA damage, and senescence. In the same vein, the silencing of XPF and XPG mitigates Tax senescence, while deficiency in either or both frequently occurs in ATL of all types. ATL cells maintain constitutively active NF-κB, accumulate R-loops, and resist Tax-induced senescence. These results suggest that ATL cells must have acquired adaptive changes to prevent senescence and benefit from the survival and proliferation advantages conferred by Tax and NF-κB. In this review, the roles of R-loops in Tax- and NF-κB-induced DNA DSBs, senescence, and ATL development, and the epigenetic and genetic alterations that arise in ATL to reduce R-loop-associated DNA damage and avert senescence will be discussed.

## 1. Introduction

Human T-cell leukemia virus type-1 (HTLV-1) is a complex human delta retrovirus that causes an aggressive CD4+ T-cell malignancy called Adult T-cell Leukemia/Lymphoma (ATL) [1,2]. HTLV-1 is endemic in southwestern Japan, Africa, South America, the Caribbean Islands, and Central Australia and infects approximately 10–20 million people worldwide [2]. The majority of HTLV-1-infection is asymptomatic. However, 3–5% of infected individuals develop ATL after decades of clinical latency. ATL is classified as the aggressive acute and lymphomatous subtypes and the indolent chronic and smoldering subtypes [3]. HTLV-1 infection also leads to inflammatory and immune-mediated diseases, including HTLV-1-associated myelopathy/tropical spastic paraparesis (HAM/TSP) [4,5], uveitis, arthritis, conjunctivitis, dermatitis, and susceptibility to helminthic and bacterial infections. More recently, HTLV-1 has been associated with bronchiectasis in the indigenous people of Central Australia [6,7].

HTLV-1 is transmitted by cell-to-cell contact between infected T-lymphocytes and uninfected target cells. Human-to-human transmission occurs via the transfer of virus-infected cells through breastfeeding, transfusion of cell-containing blood or blood products, organ transplantation, and sexual intercourse. In infected individuals, HTLV-1 proviral DNAs integrate into host chromosomes. Latently infected cells are maintained by mitotic expansion. *De novo* infection within infected individuals occurs continually [8], likely due to intermittent viral reactivation and spread to uninfected cells.

The HTLV-1 proviral DNA is approximately 9 kb in size and is flanked by 5′- and 3′- long terminal repeats (LTR). The 5′ side of the proviral DNA harbors retroviral genes that encode structural and enzymatic proteins (gag, pol, env). In contrast, the 3′ pX region contains several overlapping open reading frames (ORFs) that encode regulatory proteins (p12, p13, p30, Tax, and Rex) [9]. The anti-sense strand of the HTLV-1 proviral genome encodes another regulatory protein called HBZ, HTLV-1 basic domain leucine zipper protein, whose coding sequence resides primarily between the *env* and *pX* regions [10].

Tax and HBZ are regulatory proteins that play indispensable roles in the HTLV-1 viral life cycle and ATL development. Tax is a weak or conditional DNA binding protein. It drives LTR-mediated viral mRNA transcription by forming protein complexes with the basic domain-leucine zipper transcription factors, CREB and ATF-1, to assemble on three composite enhancer DNA elements in the U3 region of the LTR. LTR-bound Tax, in turn, recruits transcriptional co-activators including CBP/p300 to promote robust viral trans-activation [9]. Tax also exerts pleiotropic effects on cell signaling. It potently activates TAK1, IKK-NF-κB, JNK, p38 kinase, and the mTOR pathways to facilitate viral replication and cell survival [11]. Curiously, Tax is also a potent clastogen that induces DNA DSBs and micronuclei formation [12] and inhibits DNA damage repair [13], activities associated with senescence induction [9].

ATL exhibits extensive genomic instability and chromosomal abnormalities compared to other lymphoid malignancies. The whole-genome and exome sequencing of 426 ATL patients had previously identified, on average, 7.9 point mutations/10^6^ bases of ATL DNA and 59.5 structural variations per ATL genome [14]. The genomic instability of ATL is thought to be caused by Tax. Tax expression is lost or silenced in approximately 50% of ATL cells due to nonsense mutations or deletions in the Tax coding sequence or deletions or DNA methylation in the 5′ LTR [15]. In Tax-null ATL cells, gain-of-function somatic mutations in the T/B cell receptor signaling pathways supplant Tax to drive NF-κB activation [14].

HBZ antagonizes many activities of Tax, including LTR and NF-κB activation [16]. It is crucial for establishing and maintaining HTLV-1 latency and the mitotic expansion of latently infected T cells. Notably, HBZ is expressed in all ATL cells and is necessary for ATL proliferation [10,17,18]. Interestingly, recent data have indicated that Tax expression occurred intermittently in ATL cell lines, including MT1, where it induced the expression of anti-apoptotic factors via NF-κB to facilitate cell survival [19]. In agreement with these results, Tax and HTLV-1 plus-strand mRNA transcripts have also been shown to express in intense bursts in lymphocytes of infected individuals cultured ex vivo [20]. Most recently, the DNA damage and senescence response induced by Tax has been associated with a nucleic structure known as an R-loop that accumulates due to NF-κB hyperactivation by Tax [21]. This review aims to integrate these recent findings and discuss the role of Tax-/NF-κB-induced co-transcriptional R-loops in ATL development.

## 2. R-Loops, DNA Double-Strand Breaks, and Genomic Instability

**Figure 1:**
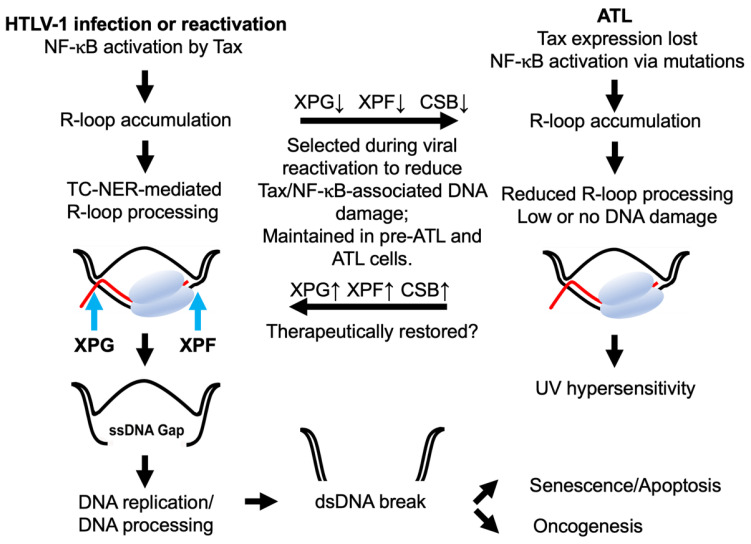
Tax-/NF-κB-induced co-transcriptional R-loops and DNA damage select for transcription-coupled nucleotide excision repair (TC-NER) deficiencies in ATL. R-loop is a three-stranded nucleic acid structure consisting of an RNA-DNA hybrid and a displaced single-stranded DNA loop. NF-κB hyperactivation by Tax leads to R-loop accumulation. During R-loop processing, XPF and XPG cleave at the 5′ and 3′ ends of the RNA-bound DNA strand to generate a single-stranded DNA (ssDNA) gap (left column). DNA replication or additional ssDNA processing leads to DSBs, resulting in genomic instability, senescence, apoptosis, or oncogenesis (bottom). Co-translational R-loops can also lead to transcription-replication conflicts and collapse of replication forks and DSBs (not depicted). Latently infected T cells deficient in TC-NER survive HTLV-1 reactivation better and evolve into ATL cells (right column). Somatic mutations in TCR signaling develop in ATL to drive Tax-independent NF-κB activation. NF-κB-induced R-loops accumulate in ATL cells without causing senescence due to the down-regulation of TC-NER mediators XPF, XPG, CSB, and other alterations. TC-NER deficiencies in ATL result in hypersensitivity to ultraviolet light (right column). Restoration of TC-NER is expected to increase R-loop excision, leading to DSBs and senescence of ATL cells. DNA double helix, RNA polymerase II, and mRNA are depicted in black, grey, and red.

An R-loop is a three-stranded nucleic acid structure consisting of an RNA-DNA hybrid and a displaced single-stranded DNA loop (Figure 1). R-loops occur naturally in the cell. They regulate immunoglobulin isotype switching, CRISPR-mediated genome editing, transcription-coupled nucleotide excision repair (TC-NER), chromatin structure, and transcription [22,23,24]. Of particular relevance here, transcriptional activation/derepression and RNA splicing/elongation/processing/export deficiencies are known to promote R-loop accumulation and genomic instability [25,26,27]. R-loop formation can occur co-transcriptionally (in *cis*, due to RNA polymerase stalling caused by excess transcriptional activation/derepression or at sites of DNA damage) or post-transcriptionally (in *trans*, as a result of RNA accumulation due to RNA processing defects or guide RNA-mediated gene targeting).

Co-transcriptionally formed R-loops have recently gained much attention as they regulate gene expression and predispose the genome to DNA damage. The non-template single-stranded DNA of the R-loop is vulnerable to DNA altering agents like activation-induced cytidine deaminase or DNA endonuclease and causes lesions or nicks. R-loops can also interfere with DNA replication to cause replication fork collapse and DNA DSBs [28]. Activation of estrogen-response element E2 by estrogen rapidly increases gene expression, R-loop accumulation, and DNA damage in human breast cancer cell lines [29]. Likewise, TATA-binding protein (TBP) overexpression caused by HRAS^V12^ upregulates genome-wide transcription and R-loop formation in immortalized human fibroblasts [30]. More recently, R-loop excision by the TC-NER endonucleases, Xeroderma pigmentosum F (XPF) and XPG, has been causally linked to the induction of DNA DSBs and genomic instability [23] (Figure 1).

## 3. Tax Hijacks RNF8 for Canonical NF-κB Activation and DNA Damage Response Perturbation

Tax is a potent activator of canonical and non-canonical IKK/NF-κB signaling, but the underlying mechanisms remained unsolved until recently. Current data indicate that Tax hijacks and aberrantly activates RING finger protein 8 (RNF8), a lysine 63 (K63) ubiquitin E3 ligase critical for signaling DNA DSB repair, and another ubiquitin ligase, linear (M1) ubiquitin assembly complex (LUBAC), to promote the assembly of K63-M1 hybrid polyubiquitin chains in the cytosol [31,32]. This signaling scaffold, in turn, recruits and activates multiple kinases, including TAK1, IKK, mTOR, JNK, p38 kinase, etc., and their downstream effectors, leading to potent activation of canonical NF-κB and other signaling pathways [11]. How Tax activates the non-canonical NF-κB pathway remains unclear to date but likely involves the stabilization and activation of NF-κB-inducing kinase NIK [33].

It should be pointed out that the impact of RNF8 dysregulation by Tax extends beyond the cytosolic activation of the kinase cascades mentioned above. Aberrant RNF8 activation by Tax in the nucleus leads to the assembly of K63-linked polyubiquitin chains that sequester DNA damage response (DDR) factors, including RNF8, BRCA1, DNA-PK, MDC1, etc., into Tax-containing pseudo-DNA damage foci [34] known as Tax speckle structures [35,36,37] that disrupt DDR signaling and interfere with repair of DNA DSBs.

## 4. NF-κB Hyperactivation by Tax Induces Cellular Senescence

NF-κB impacts immune and inflammatory responses broadly and potently. As such, cellular pathways that lead to NF-κB activation are stringently regulated by negative feedback mechanisms to ensure NF-κB is active only in a short duration [38]. As NF-κB confers survival and proliferative advantages, it is often constitutively activated in T/B cell malignancies, including ATL [14,39,40,41,42,43,44,45].

As HTLV-1 causes ATL in infected individuals and transforms T cells in culture, it was initially thought that HTLV-1 infection induces T cells to proliferate [46]. And since Tax potently activates viral transcription and IKK/NF-κB and other signaling pathways, it has been proposed that via these activities of Tax, especially NF-κB activation and transcriptional activation of interleukins and interleukin receptors, HTLV-1-infected T cells are driven to grow and divide, leading to ATL [47,48,49]. This view, however, is oversimplified and incomplete, especially in light of the discovery of HBZ. Furthermore, persistent stimulation of IKK/NF-κB by Tax comes with a price in the form of the induction of a rapid cellular senescence response (Tax-IRS) mediated by the G1/S cyclin-dependent kinase inhibitors p21^Cip1/Waf1^ (p21) and p27^Kip1^ (p27) [50,51,52]. NF-κB blockade by ΔN-I-κBα, a degradation-resistant truncation mutant of I-κBα, the inhibitor of NF-κB, dramatically prevented senescence [53].

Of note, the transcriptional activity of NF-κB is critical for Tax-induced senescence [54]. RNA silencing of p65/RelA and its upstream kinases, especially NIK and IKKα, which drive RelA activation via Ser-536 phosphorylation, mitigated senescence [53,54]. Importantly, senescence induction is not merely a result of Tax over-expression. Naive cells infected by HTLV-1 in culture arrest in senescence [55,56,57], and only infected cells expressing Tax at low levels or not at all escape senescence [56]. As expected, NF-κB inhibition allows infected cells to clonally expand [57].

Tax-induced senescence occurs after cellular passage through an aberrant cell cycle during which the S and G2 phases stall and mitosis is disrupted or impaired [50,58]. As discussed below, the senescence response induced by Tax is correlated with NF-κB- and R-loop-associated DNA DSBs. Other effects of Tax, such as cell cycle perturbation, cell cycle arrest, and apoptosis, are likely associated with the DNA DSBs caused by hyperactivated NF-κB. Finally, NF-κB is constitutively active in ATL cells of all types. As expected, expression or re-expression of Tax in ATL cells no longer induces senescence. These results suggest that ATL cells must have acquired genetic/epigenetic changes that can prevent or mitigate Tax-IRS [59]. Importantly, many ATL cells continue to accumulate R-loops in great abundance, raising the possibility that corrections of the said genetic/epigenetic changes may restore Tax-/NF-κB-/R-loop-associated DNA damage and senescence in ATL cells.

## 5. Transcription-Coupled Nucleotide Excision Repair and Transcription-Replication Conflict May Underlie the R-Loop-Associated DNA Double-Strand Breaks Caused by Tax

Transcription-coupled nucleotide excision repair (TC-NER) pathway is conserved and ubiquitous in organisms ranging from unicellular bacteria and yeast to mammals. It consists of a multiprotein repair system capable of recognizing and processing DNA structural distortions and lesions induced by UV irradiation, reactive oxygen and nitrogen species, and mutagens that introduce bulky chemical adducts in DNA. TC-NER pathway repairs DNA damage by a “cut and patch” mechanism. Initiation of repair occurs upon the physical blockage of RNA polymerase II (RNAPII) at the sites of DNA lesions/distortions during transcription. RNAPII stalling, in turn, gives rise to an R-loop formed by the nascent RNA transcript, the stalled RNAPII, the DNA template containing the lesion, and the single-stranded complementary DNA strand that loops out (see Figure 1). TC-NER endonucleases XPF and XPG are then recruited to the 5′ and 3′ sides of the R-loop to excise the DNA lesion and the short (24–32 nt) RNA-DNA hybrid from the damaged template strand. Gap-filling DNA repair then occurs using the undamaged DNA strand as a template [60]. Autosomal recessive genetic disorders such as Xeroderma Pigmentosum (XP) and Cockayne Syndrome (CS), are caused by mutations in key mediators of the TC-NER pathway, including XPF, XPG, CSB, etc., that disable the repair of UV-induced DNA damage [61], causing extreme sensitivity to sunlight and increased risk of cutaneous neoplasms.

When R-loops accumulate due to Tax-induced NF-κB hyperactivation, they are thought to be excised much like those that form co-transcriptionally at UV-induced DNA lesions, leading to single-stranded DNA gaps. DNA replication or additional DNA incision then gives rise to DNA DSBs. Indeed, NF-κB blockade prevents Tax-induced R-loop accumulation, DNA damage, and senescence [21,53]. The silencing of XPF and XPG also mitigates Tax senescence, while deficiency in either or both frequently occurs in ATL cells of all types, resulting in sensitivity to UV irradiation [21]. Finally, many Tax-expressing cells progress through the S, G2, and M phases of the cell cycle with difficulties. This aberrant cell cycle is accompanied by a dramatic rise in the mRNA and protein levels of p21 and p27, leading to cellular senescence [50]. Notably, the increase in p21 begins in the S phase, persists through G2 and M phases, and ends with p21 and p27 levels reaching their peaks in an irreversible G1/senescence arrest accompanied by chromosomal abnormalities [50,58]. Excess co-transcriptional R-loops can also cause transcription-replication conflicts (TRC) and jamming of replication fork progression to cause replication fork collapse and DNA DSBs [24]. Whether TRC plays a role in Tax-induced DNA DSBs and senescence induction remains to be demonstrated.

## 6. NF-κB Hyperactivation and Excess R-Loop Accumulation in ATL Cells

Excess R-loop accumulation threatens genomic integrity and contributes to oncogenic, neurodegenerative, and inflammatory disorders [62,63,64,65]. As discussed in a recent review [64], several cellular factors are known to regulate the homeostasis of R-loops. Notable among them are (i) RNaseH1 and RNaseH2, ribonucleases that specifically degrade the RNA moieties in R-loops; (ii) RNA/DNA helicases like Sen1 in yeast, and Senataxin (SETX) and DHX9 in humans; (iii) DNA topoisomerases that relax DNA-negative supercoiling induced by R-loops; (iv) mRNA biogenesis factors that suppress R-loop formation; and (v) chromatin remodeling factors such as the FACT complex; (vi) BRCA1, BRCA2, and members of the Fanconi anemia pathway that directly or indirectly remove or resolve the R-loops that block DNA replication.

The survival and proliferative advantages conferred by NF-κB for ATL cells outweigh the disadvantages associated with R-loops. NF-κB is constitutively active in ATL cells, and, as expected, R-loop levels therein are significantly elevated (2–3 fold of non-ATL control) [21]. These data raise the questions of what adaptive changes have evolved in ATL cells to allow NF-κB and Tax to be exploited for cell survival and proliferation without triggering extensive DSBs and senescence/cell cycle arrest and whether senescence can be reinstated by reversing such changes.

Do ATL cells mitigate the risk of excess R-loops by preventing their formation or resolving them via the cellular factors described above? Transcriptional repression of RNF8, the K63 ubiquitin E3 ligase hijacked by Tax for canonical NF-κB activation, is common in ATL cells of all types [34]. Many ATL cells also frequently down-regulate TC-NER factors such as XPF and XPG [21]. These alterations can reduce NF-κB-associated R-loop accumulation and moderate R-loop excision to lessen DNA DSBs. A deep dive into the transcriptomic and genomic data of ATL may reveal additional clues.

Other changes in ATL cells likely prevent the senescence response mediated by p21 and p27. In this vein, it is interesting to note that Kaposi’s sarcoma-associated herpesvirus (KSHV) viral cyclin (vCyclin) forms a vCyclin-CDK6 complex that resists p21 and p27 inhibition and targets p27 for degradation to drive cell proliferation [66,67]. Indeed, KSHV vCyclin effectively prevents the senescence response/G1 arrest induced by Tax and KSHV vFLIP, the KSHV-encoded activator of IKK/NF-κB. Remarkably, vCyclin and vFLIP are co-expressed in a bi-cistronic KSHV latency-associated mRNA transcript [51], consistent with their functional co-dependency. Thus, it would not be surprising if some of the key adaptive changes in ATL up-regulate G1 and G1/S cyclins expression and CDK4/6 activities to overcome the p21- and p27-mediated cell cycle blockade integral to Tax-/NF-κB-induced senescence/cell cycle arrest.

## 7. NF-κB-Induced R-Loops and DNA Double-Strand Breaks in ATL

R-loop accumulation is often a result of aberrant mRNA transcription, processing, or export. [23,68]. Excess mRNA transcription that results from potent NF-κB activation by Tax during viral replication is expected to promote R-loop accumulation and DNA DSBs. Of note, many ATL-significant and indel (insertion and deletion)-containing genes, including B2M, FAS, GATA3, HLA-B, IL-10, NFKBIA, TNFAIP3, and TP53, are transcriptionally activated by Tax via NF-κB. These genes encode proteins that are critical for the negative feedback regulation of the IKK/NF-κB pathway (TNFAIP3 and NFKBIA), tumor suppression (FAS and TP53), and host immune responses (GATA3, IL-10, B2M, and HLA-B). It is tempting to speculate that the indels within these genes originated from non-homologous end joining (NHEJ)-mediated repair of R-loop-induced DNA DSB and emerged as a result of recurrent viral reactivation. R-loop-induced DSBs may also accelerate the genetic exchange between mutant and wild-type alleles via homology-directed repair, leading to loss of heterozygosity and inactivation of tumor suppressor genes. Finally, if indeed Tax-/NF-κB-induced R-loops drive the genomic instability of ATL, why do only a select few among the approximately 500 genes under the transcriptional regulation of NF-κB incur indels and become significant for ATL development? Are they positively selected to favor ATL during the long disease course? Are there more indels in NF-κB-regulated genes of ATL than meet the eye?

## 8. Concluding Remarks and Future Direction

HTLV-1 infection in cell culture causes most cells to become senescent or senescent-like due apparently to the cytopathic effect of Tax-related DNA damage. Only a minor fraction of infected cells that do not express Tax or express it at low levels manage to survive and persist [56]. The fates of primary CD4+ T cells freshly infected by HTLV-1 in vivo remain unknown. It is well established that infected CD4+ T cells in HTLV-1 carriers show no detectable viral mRNA or protein expression, and a robust CTL response against Tax keeps viral replication in check [69]. Infected cells undergo limited oligoclonal expansion, likely due to HBZ action, giving rise to reservoirs of T cells harboring latent proviral DNA genomes that persist in the infected host and serve as the vehicles of HTLV-1 transmission and pathogenesis. A long-term follow-up of HTLV-1 carriers in Japan had previously demonstrated increased proviral DNA loads (PVLs) preceded the onset of ATL. PVL increase was coupled with the expansion of preleukemic clones that appeared as early as eight years before disease diagnosis [70]. A longitudinal study of Jamaican children who became HTLV-1-infected perinatally also showed that *de novo* HTLV-1 infections continued to occur. And clones that appeared soon after the initial infections persisted and, on rare occasions, underwent significant expansion [8].

**Figure 2:**
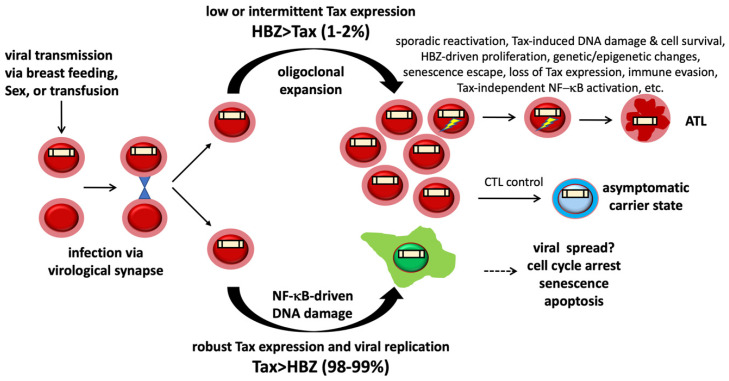
HTLV-1 and ATL development HTLV-1 infection is cell-mediated and leads to either active viral replication and senescence or latency with low or intermittent Tax expression. HBZ, in turn, stimulates CD4+ T cells latently infected by HTLV-1 (LICs) to expand mitotically. A robust cytotoxic T lymphocyte (CTL) response against Tax keeps HTLV-1 replication in check in most virus carriers. LICs constitute the cell reservoir from which ATL emerges. Intermittent HTLV-1 reactivation (denoted by flash signs), Tax expression, NF-κB activation, and R-loop accumulation drive DNA damage and genomic instability in LICs. Recurrent viral reactivation also selects for epigenetic and genetic changes that mitigate/prevent the DNA damage/senescence response induced by Tax and NF-κB and facilitate the proliferation of ATL cells.

Recent evidence demonstrates that latent HTLV-1 provirus reactivates sporadically and expresses Tax in a short, intense burst of ~19 h to induce anti-apoptotic factors that enhance cell survival. Tax inhibits DNA damage response [13,34], inactivates p53 functionally [71,72], and promotes R-loop-associated DNA DSBs and genomic instability [21]. It is conceivable that intermittent Tax expression during viral reactivation drives R-loop-induced DSBs and genomic instability, giving rise to indels and other structural alterations that confer survival and proliferation advantages and are perpetuated under the influence of HBZ (Figure 2). With frequent recurrence, this process likely leads to ATL-significant mutations that inactivate specific tumor suppressors (FAS and TP53), feedback inhibitors of IKK/NF-κB (TNFAIP3 and NFKBIA), and host immune responses (B2M and HLA-B) under NF-κB control (Figure 2).

Repeated cycles of viral reactivation likely also select for subclones harboring genetic or epigenetic alterations (such as down-regulation of RNF8, XPF, XPG; and up-regulation of the functional equivalents of KSHV vCyclin, etc.) that mitigate R-loop-associated DNA DSBs and prevent senescence induction by Tax and NF-κB during reactivation, setting the stage for the acquisition of gain-of-function mutations that promote Tax-independent NF-κB activation. Identifying and targeting these alterations can reinstate R-loop-induced DNA damage and senescence (or apoptosis) in NF-κB-addicted ATL cells.

## Data Availability

As presented.

## Glossary

ATF-1	activating transcription factor 1
ATL	adult T cell leukemia
B2M	β2 microglobulin
BRCA1	breast cancer gene 1
BRCA2	breast cancer gene 2
CBP	CREB-binding protein
CREB	cAMP response element-binding protein
CSB	cockayne syndrome B
CTL	cytotoxic T lymphocyte
DDR	DNA damage response
DHX9	DEAH-box helicase 9
DNA DSB	DNA double-strand break
DNA-PK	DNA-dependent protein kinase
FACT complex	facilitates chromatin transactions complex
FAS	FS-7-associated surface antigen, a cell surface death receptor
GATA3	GATA binding protein 3
HBZ	HTLV-1 basic domain leucine zipper protein
HLA-B	human leukocyte antigen (HLA) complex-B, major histocompatibility complex class I-B
HRAS	Harvey rat sarcoma oncogene
HTLV-1	Human T-cell leukemia virus type 1
Indel	Insertion and deletion
IKK	Inhibitor of NF-κB kinase
JNK	c-Jun N-terminal kinase
LUBAC	linear (M1) ubiquitin assembly complex
MDC1	mediator of DNA damage checkpoint 1
mTOR	mammalian target of rapamycin
NHEJ	non-homologous end joining
NIK	NF-κB-inducing kinase, mitogen-activated protein kinase kinase kinase 14
NF-κB	nuclear factor kappa B
NFKBIA	NF-κB Inhibitor α
PVL	proviral load
RNAPII	RNA polymerase II
RNF8	RING finger protein 8
Sen1	yeast homolog of senataxin
SETX	senataxin, DNA/RNA helicase that resolves R-loops
TAK1	TGFβ-activated kinase 1, mitogen-activated protein kinase kinase kinase 7
Tax	Trans-activator of the X region
Tax-IRS	Tax-induced rapid senescence
TC-NER	transcription-coupled nucleotide excision repair
TNFAIP3	TNFα-induced protein 3
TP53	tumor protein p53 (or p53)
XPF	xeroderma pigmentosum F
XPG	xeroderma pigmentosum G
